# The Impact of Physical Performance on Functional Movement Screen Scores and Asymmetries in Female University Physical Education Students

**DOI:** 10.3390/ijerph18168872

**Published:** 2021-08-23

**Authors:** Dawid Koźlenia, Jarosław Domaradzki

**Affiliations:** Department of Biostructure, Faculty of Physical Education & Sport, University School of Physical Education in Wroclaw, al. I.J. Paderewskiego 35, 51-612 Wroclaw, Poland; jaroslaw.domaradzki@awf.wroc.pl

**Keywords:** movement quality, physical performance, strength, power, flexibility, women, physical activity

## Abstract

Association between physical performance and movement quality remains ambiguous. However, both affect injury risk. Furthermore, existing research rarely regards women. Therefore, this study aimed to assess the impact of physical performance components on FMS scores and asymmetries among young women—University Physical Education Students. The study sample was 101 women, 21.72 ± 1.57 years, body mass index 21.52 ± 2.49 [kg/m^2^]. The FMS test was conducted to assess the movement patterns quality. Physical performance tests were done to evaluate strength, power, flexibility. Flexibility has the strongest correlation with FMS overall (r = 0.25, *p* = 0.0130) and single tasks scores. A higher level of flexibility and strength of abdominal muscles are associated with fewer asymmetries (r = −0.31, *p* = 0.0018; r = −0.27, *p* = 0.0057, respectively). However, the main findings determine that flexibility has the strongest and statistically significant impact on FMS overall (ß = 0.25, *p* = 0.0106) and asymmetries (ß = −0.30, *p* = 0.0014). Additionally, a significant effect of abdominal muscles strength on FMS asymmetries were observed (ß = −0.29, *p* = 0.0027). Flexibility and abdominal muscles strength have the most decisive impact on movement patterns quality. These results suggest possibilities for shaping FMS scores in young women.

## 1. Introduction

The physical performance and movement patterns quality affect injury risk [1,2]. Physical performance is defined as a body function that an appropriate test can objectively measure. It is a multidimensional concept which involves musculoskeletal system function, cardiorespiratory and nervous system. Physical performance is expressed by a level of single components such as strength, flexibility, speed, or endurance. [3,4]. Movement patterns quality is mainly examined by the FMS test, which detects dysfunctional movement patterns [5]. The relationships between the quality of movement patterns and physical performance components have been investigated. However, these associations have not been clearly defined. The studies published so far have focused mainly on men and mixed groups. Therefore, it is needed to establish these associations among women.

The tool for assessing the quality of the movement patterns is the Functional Movement Screen (FMS), which allows for a comprehensive evaluation of the functional state of the movement apparatus and to identify dysfunctional movement and differences in paired tests to identify asymmetries [5]. Numerous studies have indicated associations of low FMS scores with more injuries among men and women as well [6,7,8,9] and the possibility of injury prediction based on FMS score [10]. Mokha et al. [9] and Chalmers et al. [11] also demonstrated the relationship between asymmetries in the FMS test and injuries, studying young athletes. However, these results should be treated with some caution. In a replication study by Chalmers et al. [12], no similar links were observed.

The attempts to determine the relationship between the quality of movement patterns and physical performance components indicate their existence [13]. However, the direction and strength of these relationships are unclear. Especially possible associations remain ambiguous among women due to a low number of studies regarding females. Parchmann and McBridge [14], in a mixed group, and Lockie et al. [15] among female team sports athletes, did not show any links between the quality of the movement patterns, speed, and agility. These reports are opposed to Koźlenia et al. [16], who showed that better quality of the movement patterns is associated with better speed and agility tests outcomes among men. Support for these results can be found in the studies by Campa et al. [17]. Chang et al. [18] indicated the relationship between a trunk stability push-up with agility t-test result. Sannicandro et al. [19] showed a connection between the FMS score and the power of the lower limbs among professional footballers, showing that better quality of movement patterns was associated with greater power of the lower limbs. Chimera et al. [20] established strong relationships between flexibility and the trunk muscles’ strength with movement patterns quality. Similar observations also provide studies by Silva et al. [21,22] which showed the strength of the trunk muscles as a factor determining the quality of the movement patterns. However, the studies mentioned above [14,16,17,18,19,20,21,22] regard men and mixed groups, not focusing only on women and possible sex differences in associations between FMS score and asymmetries with physical performance components. However, Kibler et al. [23] proved that women are characterized by greater flexibility compared to men, who, in turn, have greater strength than women. The above observations could translate into relationships between the results of physical performance tests and the movement patterns quality and cause the sex differences in the single motor tasks scores in the FMS test described by Schneiders et al. [24]. They showed that men performed better than women in the trunk stability push-up (TSPU) and rotary stability (RS). Miller and Susa [25] noted a similar observation.

In the light of this observation, there is a need to keep in mind that physical performance and movement quality have an influence on injury risk [1,2]. Therefore, their interconnectedness should be explored. However, published studies mostly regard men and mixed groups in the literature, not only on women. Thus, this study aims to assess the impact of physical performance components on FMS scores and FMS asymmetries among young women—University Physical Education Students. Specifically, it was also examined the simple association between physical performance tests and FMS scores. These observations let to described which physical performance components are crucial to improving the quality of movement patterns, what can positively affect physical fitness and reduce injury risk.

## 2. Materials and Methods

### 2.1. Study Sample

The study sample consisted of 101 young adult women whose average age was 21.72 ± 1.57 years. All subjects were volunteers recruited from students at the University School of Physical Education in Wroclaw, Faculty of Physical Education and Sport. The average body weight was 60.54 ± 9.05 [kg], body height 1.68 ± 0.07 [m], and body mass index 21.52 ± 2.49 [kg/m^2^]. Subjects averagely declared 5.04 ± 3.56 h per week of physical activity. The inclusion criteria were no injuries before six weeks of the start of the study and no other medical contradiction for physical activity. All participants were fully informed about the purpose, type, research methodology, and participation conditions. They could withdraw from the research at any time without giving any reason.

### 2.2. Measurements

We followed the methods described in the study by Koźlenia and Domaradzki [2]. Participants were informed to avoid any physical activity directly before the measurements and tests. The measurements were performed in the Biokinetics Research Laboratory of the University School of Physical Education in Wroclaw (Quality Management System Certificate PN-EN ISO 9001: 2009; No. PW-48606-10E).

A SECA model 764 height and weight measuring device (SECA manufactured, Hamburg, Germany. Quality control number C-2070) was used to measure body height and weight. Based on the obtained values, the index of relative body mass BMI (kg/m^2^) was calculated according to the formula: BMI = body weight [kg]/body height [m^2^].

The quality of the movement patterns was assessed using the Functional Movement Screen (FMS). The FMS test is a battery of seven movement tasks that make up the entire test: Deep Squat (DS), Hurdle Step (HS), (In-Line Lunge (IN-L), Shoulder Mobility (SM), Active Straight Leg Raise (ASLR), Trunk Stability Push-up (TSPU), Rotary Stability (RS). The tests were performed with a standard FMS kit (Functional Movement Systems, Inc, Chatham, MA, USA). According to Cook et al. [5], no warm-up directly before the test was performed. Single motor tasks are rated on a scale of 0 to 3 according to clear guidelines described for each test [5]. The maximum score is 21 points. From 14 points and below, the risk of injury increases significantly [26].

Handgrip strength of the upper limbs was examined using a hydraulic dynamometer with an adjustable handle SAEHAN SH5001 (Saehan Corporation, Masan, South Korea). The measurements were done with an accuracy of 1 kg. The subject keeps his arm lowered so that the upper limb does not touch the body. Holding the dynamometer tightly their hand, hand clenching was performed with maximum force for about 2 s. Two attempts were made for each limb. The best result on both limbs was considered.

A long jump test was used to assess lower limbs power. From two made attempts, the better result was considered. Jump length was measured from the back of the heels. The measurement was performed with an accuracy of 0.5 cm. The subject performed the test from the established line, made a jump from both lower limbs with a swing of the upper limbs landing on both legs.

The strength of the abdominal muscles was tested with the sit-ups test. The test consists of making as many sit-ups as possible within 30 s. One attempt was made. The subject was laid down with the lower limbs bent at the knee joints at an angle of 90°. The feet were blocked. The subject began the test by lying down with her hands clasped behind the nape of her neck, performing torso bends and touching knees with elbows.

Flexibility was measured using the sit-and-reach test. The measurement was performed with an accuracy of 0.5 cm. The examined person sat down with the lower limbs straightened in the knee joints by placing feet against the sidewall of the table. While maintaining the extension in the knee joints, the subject bent forward and tried to move the ruler on the table as far as possible along the scale. The tests scores were measured from the 0 cm point. The measurement was performed with an accuracy of 0.5 cm. Of the two trials, the better result was considered.

### 2.3. Statistics

The means, standard deviations, and confidence intervals were calculated for the data meeting the assumptions of normality of the distribution or the median, and standard errors for the data that did not meet the assumptions of the normal distribution. Spearman’s rank correlation was calculated to investigate the strength and direction of relationships between the quality of the movement patterns (FMS scores) and physical performance tests. Multiple regression was used to determine the impact of the physical performance components on FMS overall and asymmetries. The significance level α = 0.05. Statistica v13.3 from Statsoft Polska (Cracow, Poland) was used for statistical analyses.

## 3. Results

Table 1 includes descriptive statistics for physical performance tests results.

Table 2 shows the FMS overall score, single tasks score, and asymmetries numbers in the bilateral test. The mean FMS overall score is 14.96 ± 2.21, and a median of 15 points indicates the study sample has high-quality movement patterns.

Spearman’s correlation for FMS overall, single tasks score, and asymmetries number revealed the higher sit-and-reach test result is associated with the better FMS overall r = 0.25, *p* = 0.2130 and lower number of FMS asymmetries r = −0.31, *p* = 0.0018, better hurdle step score (HS) r = 0.21, *p* = 0.0357, shoulder mobility score (SM) r = 0.34, *p* = 0.0133, and asymmetries r = −0.23, *p* = 0.2010, active straight leg raise score r = 0.50, *p* > 0.0001 and asymmetries r = −0.27, *p* = 0.0055. Additionally, the higher sit-ups test results were associated with the lower number of FMS asymmetries r = −0.27, *p* = 0.0057, and in-line lunge asymmetries r−0.25, *p* = 0.0126. No other statistically significant correlation was observed.

**Figure 1:**
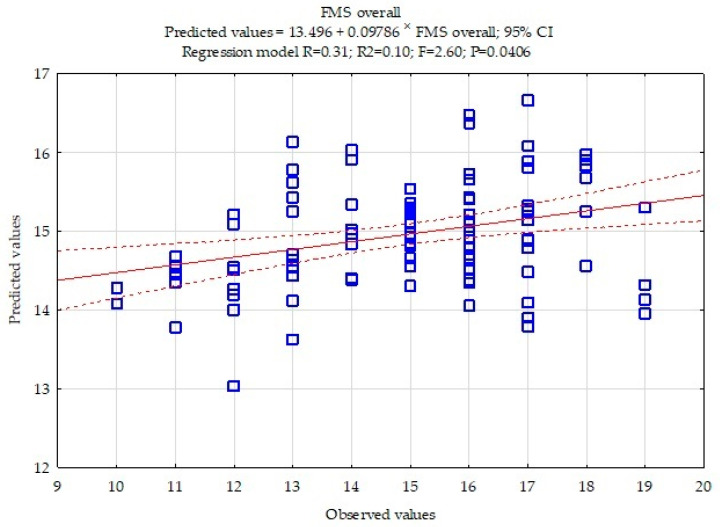
Multiple regression model for FMS overall score.

The multiple regression model for FMS overall is presented in Figure 1. The model is statistically significant, *p* < 0.0406.

Multiple regression results for FMS overall score are included in Table 3. Flexibility had the strongest statistically significant impact on FMS overall ß = 0.25, *p* = 0.0206. An increase in the sit-and-reach test score by 1 cm is associated with improving FMS overall score by 0.08 points.

**Figure 2:**
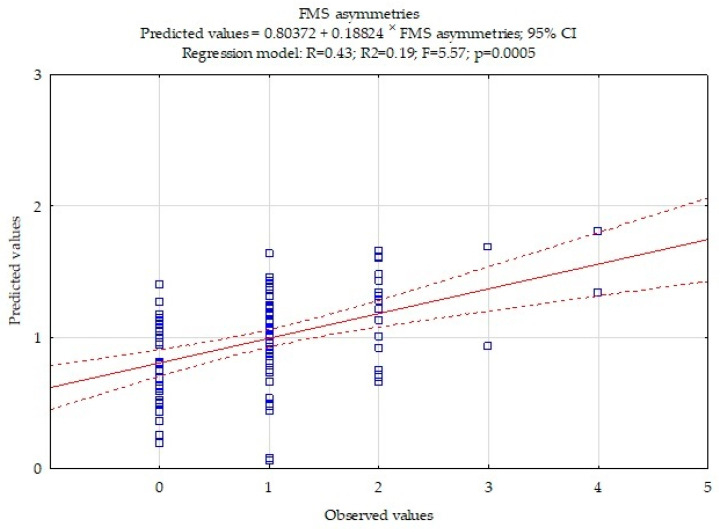
Multiple regression model for FMS asymmetries number.

The multiple regression model for FMS asymmetries is presented in Figure 2. The model is statistically significant, *p* < 0.0005.

Multiple regression results for FMS asymmetries numbers are included in Table 4. Flexibility had the strongest statistically significant impact on FMS asymmetries ß = −0.30, *p* = 0.0014. Additionally, FMS asymmetries depend on abdominal muscles strength ß = −0.29, *p* = 0.0027. An increase in the sit-and-reach test score by 1 cm is associated with reducing asymmetries by 0.04. An increase in the sit-ups test by one rep reduces FMS asymmetries number by 0.05.

## 4. Discussion

The quality of movement patterns and the level of physical performance affect the risk of injury, which raises further questions about their relationship [1,2]. There have been attempts in the literature to answer this type of ambiguity. However, some differences in observation do not allow clear conclusions on this matter, especially considering women due to the low number of studies among females.

Our results showed that flexibility and abdominal muscle strength have an influence on movement patterns quality among young women. The strength of abdominal muscles is crucial in trunk stability, whereas a good level of flexibility aids the optimal range of motion in joints [27,28]. Both mentioned physical performance components allow movement without restrictions in joints with simultaneous stabilization in various body positions, thus avoiding compensations that disturb the movement patterns. [29]. Our results confirm this approach that is supported by the literature which provides related observations [20,21,22].

Most researchers agree that sex is not a factor that differentiates the overall score of the FMS test within one research group. Schneiders et al. [24] and Miller and Susa [25] did not show a statistically significant difference in the mean FMS score between physically active men and women. On the other hand, diversity can be seen in comparisons between groups from different research studies. The type of physical activity may explain the differences in FMS scores between groups from other studies. This can be seen when comparing the FMS scores results among various sports groups (e.g., footballers [30], volleyball players [31], or runners [8]). The quality of movement patterns can be shaped by appropriate training [32,33].

In the case of assessments of the FMS single motor tasks, sex differences are observed. It was shown that men performed better in a test that required stability and strength (TSPU and RS), whereas women achieved higher scores in a mobility test (SM and ASLR) [24]. A study by Miller and Susa [25] confirms this observation, which indicated that women achieved better results in shoulder mobility (SM) and active straight leg raises (ASLR), while men had higher scores in the trunk stability push-up (TSPU) and in-line lunge (IL-L). Anderson et al. [34] also indicated that women performed a weaker trunk stability push-up (TSPU). Similar observations were made in the study by Chimera et al. [20]. Women achieved better results in tasks requiring flexibility, active straight leg raise (ASLR), and shoulder mobility (SM), whereas men achieved better results than women in trunk stability push-up (TSPU) and rotary stability (RS). These results indicate that, despite averagely comparable results in FMS overall score between sexes, its structure could be differentiated by scores in single tasks. It could be associated with sex differences in physical performance [23]. Therefore, it suggests a need to consider sex differences in targeting the development of physical performance to improve movement quality.

The median value of the FMS overall score of our study sample was 15, indicating high-quality movement patterns associated with low injury risk [26]. Literature shows that high-quality movement patterns characterize young, physically active women. Chimera et al. [6] revealed that female athletes’ average FMS overall score was above 14 points. A similar observation was added by Anderson et al. [34], which showed the mean FMS score among females was above 15. Our study sample performed better in the standing long jump than nonathletes females [35]. In the case of the sit-and-reach test, our study sample achieved comparable results to the female athletes from the study by Lopez-Minaro et al. [36]. In the handgrip test and 30 s sit-ups test, our study sample achieved better results than average populations [37,38].

Analyzing the relationship between the quality of movement patterns and physical performance components indicates a high, statically significant correlation between the sit-and-reach test score (flexibility), the FMS overall, and the FMS asymmetries number. The association in single tasks score was observed as an active straight leg raise (ASLR), shoulder mobility (SM), and asymmetries in these tests. A better result in the sit-and-reach test score was also associated with a better hurdle step (HS) score. Additionally, a higher level of flexibility related to a lower number of asymmetries was also observed in better results in the sit-ups test, suggesting that strong abdominal muscles are essential in maintaining functional symmetry. Other physical performance tests showed low and statistically insignificant correlations, suggesting no linear correlation between physical performance tests, such as handgrip or long jump, and the FMS scores. Similar observations were noted in other studies, especially regarding the relationship between flexibility and the quality of movement patterns. However, literature has limited observations considering the association between movement quality and FMS scores with physical performance components such as flexibility or strength. Therefore, it is hard to refer our results to only female groups. Some references must be made to mixed and male groups. Grygorowicz et al. [39] observed that female soccer players have better quality of movement patterns with a higher level of flexibility, which confirms our observations. Glass et al. [40] showed associations between the higher level of strength, balance, and flexibility with the quality of the movement patterns. In mixed groups, Jenkins et al. [41] observed that a better range of hip joint motion was associated with higher quality of movement patterns. Similarly, Yildiz et al. [42] indicated improved FMS scores and flexibility among children during tennis training after using a training intervention. Song et al. [43] noted that flexibility development was associated with improved FMS scores in males. In a study of male baseball players, Liang et al. [44] showed associations of FMS scores with the flexibility of the rectus femoris and speed abilities. Chimera et al. [20] showed that worse ranges of motion in the hip and knee joints negatively affect the movement patterns quality in a mixed study sample. The same authors [18] also observed that stronger trunk muscles positively influenced the FMS scores. Surprisingly, in our research, a higher score in trunk stability push-up (TSPU) was not directly associated with a better result of the sit-ups, whereas the study by Silva et al. [20,21] found links between the trunk stability push-up (TSPU) and the physical fitness of the study group. However, handgrip strength and the FMS scores do not seem to have clear connections [20]. Sannicandro et al. [19] indicated that footballers presented better quality movement patterns that generated greater lower limb power. Willigenburg and Hewett [45] observed correlations between the length of the long jump and the overall FMS score among American football players. In our study, a correlation between the long jump test score and the FMS overall and the deep squat (DS) score was not observed in women. The conclusions drawn from the above observations indicate a clear relationship between the quality of movement patterns and physical performance components. The single motor tasks of the FMS test are specifically related to the level of selected physical performance components. In this type of analysis, there is a need to consider the possible differences between sexes. However, our study shows that flexibility has the most decisive impact on the FMS overall score among female students.

Our analysis also showed that the number of asymmetries observed during the FMS test is related to flexibility and abdominal muscle strength, indicating these factors’ significant role in shaping symmetrical movement patterns. However, the literature so far does not pay much attention to the number of FMS asymmetries concerning the level of physical fitness. Athletes with a higher number of asymmetries are 1.8 times more likely to be injured [46]. Similar observations are made by Mokha et al. [9], which also indicated a significant risk of injury due to asymmetries. Considering the relationship between flexibility and muscle strength and the risk of injury [47,48], the importance of these abilities as a key to developing high-quality movement patterns is growing.

## 5. Conclusions

In young, healthy women, flexibility and abdominal muscles strength are significantly associated with the quality of movement patterns, expressed as an FMS overall score and FMS asymmetries. Furthermore, flexibility is a component of physical performance with the most decisive impact on movement patterns quality in FMS overall score and asymmetries number, whereas abdominal muscle strength only influences on asymmetries in FMS. Our results indicate the importance of flexibility and abdominal muscle strength for movement patterns quality among young women. The appropriate range of motion in joints with abdominal muscle strength that provides trunk stability helps to avoid compensation in movement. It potentially suggests that FMS scores can be shaped throughout the development of flexibility and abdominal muscle strength. However, further studies need to verify if developing these abilities improves movement patterns quality in young women.

We are aware that our study has some limitations. The analysis could be augmented with more physical performance tests measuring other abilities, such as speed and endurance. It is also worth analyzing how the type of physical activity undertaken affects the relationship between the quality of movement patterns and physical performance. These aspects should be considered in further studies.

## Figures and Tables

**Table 1:** Physical performance tests results.

Variable	Mean	SD	CI −95%	CI +95%
Hand grip (N/kg)	36.11	5.93	34.94	37.28
Long jump (cm)	179.42	31.08	173.28	185.55
Sit-ups (reps/30 s)	24.23	4.66	23.31	25.15
Sit and reach (cm)	13.67	7.37	12.21	15.12

**Table 2:** Characteristics of the FMS scores.

Variable	Median	SE
FMS	15	0.22
DS	2	0.07
HS	2	0.06
IN-L	2	0.07
SM	3	0.09
ASLR	3	0.06
TSPU	2	0.08
RS	2	0.05
HS A	0	0.04
IN-L A	0	0.04
SM A	0	0.05
ASLR A	0	0.04
RS A	0	0.02
FMS Asymmetries	1	0.09

Abbreviations: FMS—overall score; DS—deep squat; HS—hurdle step; HS A—hurdle step-asymmetry; IN-L—in-line lunge; IN-L A—in-line lunge-asymmetry; SM—shoulder mobility; SM A—shoulder mobility-asymmetry; ASLR—active straight leg raise; ASLR—active straight leg raise-asymmetry; TSPU—trunk stability push-up; RS—rotary stability—overall; RS A—rotary stability-asymmetry.

**Table 3:** Multiple regression results for FMS overall score.

Dependent Variable	Independent Variables	ß	ß SE	B	b SE	t	*p*
FMS overall	Hand grip (N/kg)	0.11	0.10	0.04	0.04	1.08	0.2811
	Long jump (cm)	0.09	0.10	0.01	0.01	0.89	0.3781
	Sit-ups (reps/30 s)	0.15	0.10	0.07	0.05	1.49	0.1395
	Sit and reach (cm)	0.25	0.10	0.08	0.03	2.61	0.0106

**Table 4:** Multiple regression results for FMS asymmetries number.

Dependent Variable	Independent Variables	ß	ß SE	B	b SE	t	*p*
FMS asymmetries	Hand grip (N/kg)	0.04	0.09	0.01	0.01	0.42	0.6739
	Long jump (cm)	0.01	0.09	0.00	0.00	0.06	0.9553
	Sit-ups (reps/30 s)	−0.29	0.09	−0.05	0.02	−3.08	0.0027
	Sit and reach (cm)	−0.30	0.09	−0.04	0.01	−3.29	0.0014

## Data Availability

The datasets used and analyzed during the current study are available from the corresponding author on reasonable request.
